# Fractionated Anionic PAM Dosing Under High Salinity: Controlling Floc Growth and Stability

**DOI:** 10.3390/polym18010050

**Published:** 2025-12-24

**Authors:** Jahir Ramos, Eder Piceros, Tiare D. Medina, Pedro Robles, Gonzalo R. Quezada, Williams Leiva, Ricardo I. Jeldres

**Affiliations:** 1 Departamento de Ingeniería Química y Procesos de Minerales, Facultad de Ingeniería, Universidad de Antofagasta, Antofagasta 1240000, Chile; jahir.ramos.ibanez@ua.cl; 2 Facultad de Ingeniería y Arquitectura, Universidad Arturo Prat, Iquique 1110939, Chile; 3 Departamento de Ciencias de la Tierra, Facultad de Ciencias Químicas, Universidad de Concepción, Edmundo Larenas 129, Casilla 160-C, Concepción 4030000, Chile; tmedina2025@udec.cl; 4 Escuela de Ingeniería Química, Pontificia Universidad Católica de Valparaíso, Valparaíso 2340000, Chile; pedro.robles@pucv.cl; 5 Departamento de Ingeniería de Procesos y Bioproductos, Facultad de Ingeniería, Universidad del Bío-Bío, Concepción 4030000, Chile; grquezada@ubiobio.cl; 6 Facultad de Ingeniería, Universidad San Sebastián, Sede Concepción, Concepción 4030000, Chile; williams.leiva@uss.cl; 7 Advanced Mining Technology Center (AMTC), Universidad de Antofagasta, Antofagasta 1240000, Chile

**Keywords:** seawater flocculation, anionic polyacrylamide, fractionated dosing, adsorption–bridging, FBRM, size distributions, fractal dimension, aggregate density

## Abstract

The use of seawater in mineral processing poses significant challenges for solid–liquid separation, including polymer chain contraction, accelerated coagulation, and brittle aggregate formation. This study evaluates the impact of fractional dosing of anionic polyacrylamide (PAM) on the formation, structure, and sedimentation performance of flocs in quartz-kaolinite suspensions prepared in seawater. Four dosing schemes (1, 2, 3, and 4 pulses) were analyzed, maintaining a total dose of 15 g/t and flocculation times of 75, 90, and 105 s. Sedimentation assays, kinetic monitoring using FBRM, size distributions, fractal dimensions, and bulk density were integrated to characterize the aggregation process. The results show that all fractional strategies outperform single-pulse dosing, with the three-pulse scheme (0–30–60 s) standing out, achieving the highest settling rates, the most significant fines reduction, and the best structural robustness. FBRM kinetics reveal stepped growth, less shear breakage, and more stable maturation when polymer addition is divided temporally. Consistently, fractal dimension and aggregate density reach their maximum values after three 90 s pulses, indicating more compact, less porous structures. Zeta potential analysis confirms a strong polymer-particle interaction in kaolinite under high salinity. The superior performance of the multi-pulse strategy is explained by the progressive availability of active polymer segments during aggregate formation and maturation. Each pulse is incorporated into a partially structured suspension, in which unoccupied mineral surfaces and flocs from the early stages of consolidation still exist. This staggered adsorption avoids local overdosing associated with flash injections, improves bridging efficiency, reduces brittle aggregate formation, and promotes more uniform restructuring.

## 1. Introduction

The increasing scarcity of water resources in arid mining regions has driven a strategic shift in mineral processing operations, promoting the use of seawater as an alternative to freshwater [1,2]. This transition, driven by both regulatory restrictions and environmental concerns, poses significant technical challenges, particularly during solid–liquid separation. In this context, tailings thickening—a critical process for the recovery and recirculation of process water—is directly impacted by the salinity of the medium, which modifies the interfacial and rheological properties of mineral suspensions.

The use of seawater affects thickening efficiency through multiple mechanisms. On the one hand, the high ionic strength compresses the electrical double layer of fine particles, reducing repulsive forces and facilitating colloidal coagulation. On the other hand, divalent cations (such as Mg^2+^ and Ca^2+^) promote precipitation reactions, especially under alkaline conditions, generating metal hydroxides that interfere with flocculation and affect the clarity of the supernatant [3]. Furthermore, the contraction of flocculant chains in saline media reduces their effective extension in solution, compromising their bridging capacity and, consequently, the formation of robust, settleable flocs [4]. Consequently, maximizing the efficiency of flocculant reagents in this hostile environment is critical to the process’s sustainability. In clay tailings at pH 7, surface reactivity is controlled by inherent mineral differences. Quartz exhibits a uniformly negative surface, dominated by silanol groups, while kaolinite has permanently negative basal faces and pH-dependent aluminol rims with an affinity for divalent cations. In seawater, the extreme compression of the electrical double layer and the specific adsorption of Mg^2+^ and Ca^2+^ markedly modify the surface potential, especially in kaolinite [5,6]. Our zeta potential results confirm that this mineral exhibits a more intense variation with the addition of PAM, indicating a stronger polymer-particle interaction. This asymmetry implies that kaolinite largely controls the fines population and, therefore, the effective polymer demand, establishing the basis for the differential response observed under fractional dosing.

In industrial operations, particle flocculation occurs primarily in the thickener’s feedwell [7]. This component plays a fundamental role in generating the appropriate hydrodynamic conditions for contact between particles and polymer. Its interior combines turbulent mixing zones, necessary for dispersing the flocculant, with more controlled flow zones that allow aggregate growth. The quality of the flocs formed—and, consequently, the efficiency of the sedimentation process—is directly influenced by the feedwell design, the internal flow pattern, and the flocculant dosing strategy [8].

In industrial practice, flocculant dosing is performed continuously through multiple inlet points distributed around the feedwell, and even through ring-type addition systems that inject the reagent in a continuous, circular manner. These schemes aim to ensure uniform, continuous coverage of the polymer in the suspension; questions remain about the dynamics of flocculant-particle interactions and the structural growth of aggregates. Studies that analyze the hydrodynamic and physicochemical implications of these dosing methods in detail are scarce [9,10]. It is unknown to what extent the temporal or spatial fractionation of the flocculant influences the quality and stability of the flocs formed in seawater. The partially hydrolyzed PAM used (≈30% hydrolysis, 12–15 MDa) represents a critical balance between chain extension and charge density in high-salinity environments. Higher degrees of hydrolysis would increase electrostatic repulsion but would also favor shielding by divalent cations, reducing bridging efficiency. Conversely, a lower degree of hydrolysis would decrease the number of carboxylate groups available for multisite adsorption and limit loop and tail reconfiguration during consolidation. Previous studies have shown that in seawater, an intermediate degree of hydrolysis maximizes the number of active segments without completely collapsing the chain conformation, allowing for effective bridge formation and contributing to the structural stability of the aggregates. Therefore, a PAM with ~30% hydrolysis is particularly suitable for overcoming salinity-induced polymer shrinkage.

Research in biological and environmental systems has shown that this methodology can improve process efficiency. Wen et al. [11] compared the effects of single- and multiple-dosing of trivalent aluminum (Al^3+^) on flocculation and sedimentation in activated sludge systems to optimize solid–liquid separation. Their study showed that although both strategies improved flocculation, multiple dosing enabled simultaneous achievement of lower effluent turbidity and a significant reduction in the sludge volumetric index (SVI), resulting in more compact, easily settleable flocs. This behavior was attributed to the fact that fractional dosing of Al^3+^ prolongs the interaction time between the cation and the flocs, facilitating charge neutralization and compression of the electrical double layer, and decreasing the content of extracellular polymeric substances (EPS), which, in excess, deteriorate the floc structure. Kadooka et al. [12] verified through simulations and experimental tests that fractionalizing the polymer dose reduced turbidity and process variability. Zhang et al. [13] recently investigated an intermittent dosing strategy to improve the harvesting efficiency of microalgae (Chlorella sorokiniana) and compared it with traditional single-, double-, and continuous-dosing methods. Their study showed that fractional addition of a cationic flocculant (CPAM) at 15 mg/L enabled harvesting efficiency over 90%, reducing polymer consumption by 50% compared to single dosing. Floc analysis revealed that the intermittent strategy promoted the formation of larger, less compact aggregates, associated with better flocculant distribution and more effective polymer-cell interactions, facilitating both bridging and surface charge neutralization. In the field of solid–liquid separation engineering, Li et al. [14] provided a relevant experimental background by demonstrating that intermittent addition of polyacrylamide can significantly improve the operational stability of a fluidized pellet bed (FPB) reactor used in wastewater treatment. Their study addressed a common limitation of continuous polymer dosing, reduced solid–liquid separation efficiency. Using a fractional PAM dosing strategy, they achieved superior performance over 60 days of operation, increasing chemical oxygen demand (COD) removal from 53–60% to 63–75%, and total phosphorus removal to values above 90%. The intermittent addition promoted controlled breakup and refloculation cycles, allowing pellet regeneration and improving both their microstructural compactness and shear strength.

In mining, the application of this strategy has been poorly explored. At the computational simulation level, computational fluid dynamics (CFD) models coupled with population balance models (PBM) have been developed to predict the evolution of aggregate size and structure under different feedwell operating conditions [8,15,16,17]. These models have demonstrated that the internal hydrodynamic conditions of the feedwell, along with the flocculant addition scheme, directly impact the efficiency of the flocculation process. Overall, these studies emphasize the significance of controlled mixing patterns and the utilization of internal structures in the feedwell to prolong flocculant residence time, enhance particle-polymer contact, and promote the formation of denser aggregates.

In addition, the use of experimental tools, such as focused beam reflectance measurement (FBRM), has enabled real-time characterization of floc formation and breakup kinetics [18,19]. This technique, widely used in recent studies, provides detailed information on aggregate size distribution and structural stability. However, its application has primarily focused on freshwater systems under continuous dosing conditions, leaving significant gaps in its use for fractional-dosing strategies and in high-salinity environments. Indeed, one of the major current challenges lies in understanding how the physicochemical properties of the saline environment interact with polymer flocculation mechanisms. In high-ionic-strength solutions, such as seawater, anionic polymer chains experience a significant reduction in length due to the neutralization of their carboxylate groups by cations [4,20]. At the same time, salinity promotes primary coagulation by reducing the thickness of the electrical double layer, thereby leading to the formation of more compact, albeit more fragile, aggregates. Moreover, at alkaline pH, typical of flotation processes, the precipitation of compounds such as Mg(OH)_2_ and CaCO_3_ directly affects the recovery efficiency of valuable minerals in flotation and the performance of traditional anionic polyacrylamides by reducing aggregate stability in tailings thickening [21,22,23].

While fractional dosing strategies have been studied in biological and water treatment systems, their application to mineral suspensions in seawater is not straightforward due to fundamental differences in particle composition, surface chemistry, and aggregation mechanisms. In this context, this work focuses on high-salinity mineral tailings, addressing polymer-particle interactions, floc structural evolution, and fines capture in a quartz-kaolinite system representative of mining operations.

Although there is literature on flocculation in seawater, the structural characterization of aggregates formed under fractional dosing strategies in saline mineral systems remains limited. In particular, aspects such as fractal dimension, bulk density, and shear strength have been little explored under conditions simulating continuous industrial operations, restricting the design of more efficient and robust dosing schemes.

In this context, this study proposes an experimental evaluation of the effect of different fractional dosing schemes of anionic flocculants on the formation, internal structure, and sedimentation of flocs generated in synthetic tailings suspensions in seawater. Advanced techniques, such as FBRM, will be used to monitor flocculation kinetics, and structural parameters, including aggregate size, sedimentation velocity, fractal dimension, and bulk density, will be calculated.

## 2. Materials and Methods

### 2.1. Polymer, Seawater, and Solid Materials

**Figure 1:**
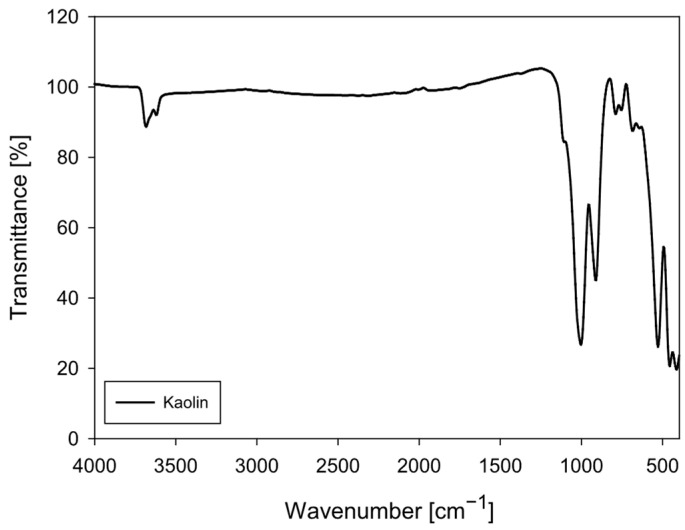
FTIR spectrum of kaolin particles (4000–400 cm^−1^).

Kaolin particles were purchased from Ward’s Science (Clay Spur, WY, USA). The FTIR spectrum of the kaolin particles (Figure 1) shows a vibrational pattern characteristic of kaolinite (Al_2_Si_2_O_5_(OH)_4_), confirming the mineralogical identity observed in the XRD analysis. Kaolinite is a 1:1 phyllosilicate whose structure incorporates internal and surface hydroxyl groups, as well as Si–O and Al–O–Si bonds, generating a set of highly specific bands in the mid-infrared. The band assignments in the obtained spectrum agree with the literature for high-purity kaolin.

In the 3700–3600 cm^−1^ region, the structural O–H stretching bands of kaolinite are clearly distinguishable, typically located at 3695, 3669, 3652, and 3620 cm^−1^. However, in this spectrum, they appear as a composite depression due to instrumental resolution. These bands correspond to tightly bound internal hydroxyls, characteristic of the mineral’s octahedral layer. A slight broadening around ~3400 cm^−1^ suggests the presence of a small amount of adsorbed water, a common feature of fine particles.

The most intense band in the spectrum appears in the 1030–1115 cm^−1^ region, attributed to the asymmetric stretching of the Si–O–Si bond, which is the main signature of the tetrahedral silicate lattice. The presence of the ~915–935 cm^−1^ band, corresponding to Al–OH stretching, is particularly diagnostic of kaolinite and confirms the presence of aluminum octahedra partially bonded to internal hydroxyl groups. Additional Si–O vibrations are observed in the 790–750 cm^−1^ and 690–660 cm^−1^ regions, while the bands between 540–470 cm^−1^ correspond to Al–O–Si and Si–O bending modes. Finally, the set of signals around 430–420 cm^−1^ represents low bending of the tetrahedral skeleton.

The absence of bands attributable to organic matter (C–H at 2920–2850 cm^−1^), carbonates (1420 and 875 cm^−1^), or other inorganic phases confirms that the sample is relatively pure kaolin with very low interference from secondary minerals. The relative sharpness of the bands suggests an ordered kaolinite, consistent with materials of good crystallinity.

**Figure 2:**
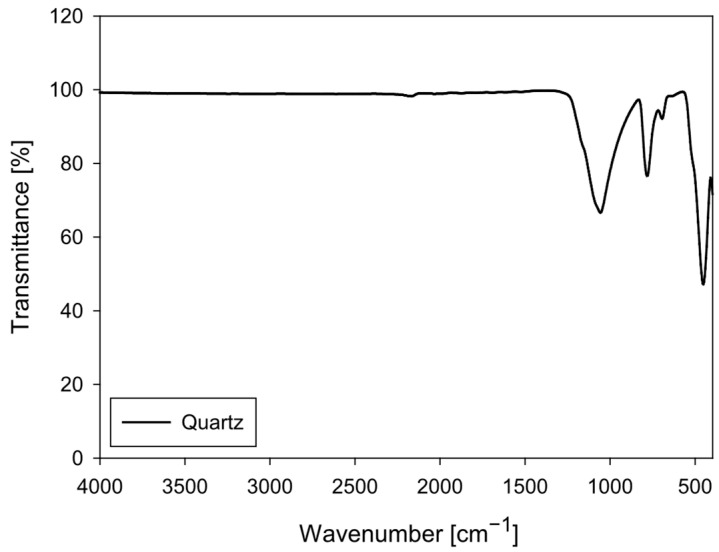
FTIR spectrum of quartz particles (4000–400 cm^−1^).

The quartz particles (SiO_2_) were obtained from Donde Capo (Santiago, Chile). The sample was sieved to obtain a particle size fraction below 270 mesh (ASTM E11). The FTIR spectrum of quartz particles (Figure 2) exhibits the characteristic vibrational bands of crystalline silica (SiO_2_), confirming that it is a highly pure, structurally ordered material. Quartz, composed of a three-dimensional network of SiO_4_ tetrahedra linked by Si–O–Si bonds, exhibits a characteristic FTIR pattern that serves as a classic reference for characterizing siliceous phases.

The most intense signal in the spectrum is observed in the 1100–1000 cm^−1^ region, attributed to the asymmetric stretching of the Si–O–Si bond, which constitutes the main band of quartz. This is followed by bands located approximately at 800–780 cm^−1^, corresponding to the symmetric Si–O stretching, and a well-defined peak around 695 cm^−1^, characteristic of the doublet associated with the symmetry of crystalline quartz. Finally, the band located in the 460–470 cm^−1^ range originates from Si–O bending, another diagnostic feature of this mineral phase.

The spectrum lacks bands in the 3700–3000 cm^−1^ region, indicating the absence of structural hydroxyl groups or strongly adsorbed water. Likewise, the absence of signals at 2920–2850 cm^−1^ (C–H), 1650–1550 cm^−1^ (C=O, N–H), and 1450–1400 cm^−1^ (–COO^−^) confirms that the sample contains no organic matter or adsorbed polymers, consistent with the fact that this quartz has not been subjected to PAM interaction.

The synthetic seawater used in the tests was prepared according to the composition described in Table 1 by dissolving ultra-high-purity reagents (Merck, Darmstadt, Germany) in distilled water.

The flocculant used in this study was a partially hydrolyzed polyacrylamide (PAM) supplied by SNF Chile S.A. (commercial name: SNF 704, Santiago, Chile). This polymer exhibits an anionic character with an average molecular weight of approximately 12–15 × 10^6^ g·mol^−1^ and a degree of hydrolysis of about 30%. The hydrolysis of amide groups to carboxylate groups creates active sites that enhance particle–polymer interactions via electrostatic attraction and bridging mechanisms. The stock solution was prepared by dissolving the solid polymer in distilled water under continuous stirring for 24 h, resulting in a 1 g/L solution. This solution was subsequently diluted to a final concentration of 0.1 g/L. All tests were performed at pH 7.

### 2.2. Surface Charge Characterization Through Zeta Potential

The zeta potential was determined to assess the particle surface charge under saline conditions relevant to adsorption mechanisms, polymer bridging, and colloidal stability. Suspensions of 1% by weight of quartz and kaolinite were prepared and adjusted to a pH of 7. Before measurements, each suspension was gently stirred for 10 min to ensure homogeneous dispersion. Measurements were performed by electrophoretic light scattering using a Litesizer 500 (Anton Paar, Graz, Austria) with an Omega cell. Data acquisition and processing were performed using Kalliope software (version 3.8.2), which applied the Smoluchowski approximation with a Henry function value of 1.5 to convert electrophoretic mobility to zeta potential.

To minimize errors resulting from transient settling or agglomeration, samples were loaded immediately after mixing and visually inspected to confirm the absence of bubbles or large aggregates. For each material, triplicate measurements were performed on freshly prepared suspensions; the reported values are averages, with standard deviations typically less than 5%.

### 2.3. Experimental Procedure for Sedimentation Tests

Synthetic tailings suspensions were prepared at 10% solids by weight, using a combination of kaolinite and quartz in a ratio of 80% Qz/20% Kao. The solid composition of 80% quartz and 20% kaolinite was selected to represent a synthetic tailings system with a clay fraction high enough to generate a significant population of fine particles and active surfaces, but not so high as to hinder the homogeneous preparation of the suspensions. This proportion falls within the typical range for Cu-Mo operations in northern Chile.

Each mixture weighed 300 g, composed of 30 g of solids and 270 g of the liquid phase. The liquid phase consisted of synthetic seawater combined with the flocculant solution, resulting in a final solids concentration of 15 g/t (g of polymer per metric ton of solids).

The mixtures were prepared in a 1000 cm^3^ cylindrical vessel equipped with a discharge valve at the base, which allowed for controlled transfer of the suspensions into 250 mL graduated cylinders for subsequent testing. Initially, the solids were added to seawater and stirred at 600 rpm for 10 min using a 30 mm diameter polytetrafluoroethylene (PTFE)-coated turbine stirrer, positioned 5 cm from the bottom of the vessel. This procedure ensured adequate particle dispersion.

The pH of the suspensions was progressively adjusted by adding lime until a pH of 7 was reached, thereby stabilizing the medium’s chemical conditions. The stirring speed was subsequently reduced from 600 to 200 rpm to prepare the suspension for controlled flocculant addition.

- Three different flocculation times were evaluated: 75, 90, and 105 s. The total flocculation times of 75, 90, and 105 s were selected because they fall within the typical residence range in a tailing thickener feedwell. Intervals that are too short result in highly porous flocs, while prolonged times promote shear deterioration. These three points represent an early stage (75 s), a stage close to optimal consolidation (90 s), and a stage before structural weakening (105 s), ensuring that all dosing strategies are applied within the first 60 s and that the remaining time reflects only the natural evolution of the system. For each flocculation time, four flocculant dosing strategies were implemented, always maintaining a total dosage of 15 g/t:
- Instant dosing: complete addition at the beginning of the process (0 s).
- Two-stage dosing: 50% addition at the beginning (0 s) and the remainder at 60 s (0–60 s).
- Three-stage dosing: additions divided into 0, 30, and 60 s (0–30–60 s).
- Four-stage dosing: successive additions at 0, 20, 40, and 60 s (0–20–40–60 s).

Once flocculation was complete, the pulp was carefully transferred to 250 mL graduated cylinders. Before beginning the sedimentation tests, each sample was subjected to three gentle manual agitations, rotating the cylinder 180° with each agitation, to homogenize the flocculant distribution without destabilizing the formed flocs. This procedure was intended to ensure more representative and reproducible sedimentation in subsequent tests.

### 2.4. Monitoring of Flocculation Kinetics Using FBRM

Synthetic tailings suspensions with a solids concentration of 10% by weight were prepared using a total mass of 300 g, composed of 30 g of solids and 270 g of liquid phase. This liquid phase consisted of synthetic seawater and the diluted flocculant solution, adjusted to achieve the required flocculant dosage.

To ensure adequate particle dispersion, the mixtures were subjected to high-speed turbulent stirring (600 rpm) for 10 min at pH 7. The Focus Beam Reflectance Measurement (FBRM) probe was subsequently inserted into the suspension, and the stirring speed was reduced to 200 rpm. This procedure maintained a constant, homogeneous solids concentration across all samples during controlled flocculant incorporation.

Three different flocculation times were evaluated: 75, 90, and 105 s. For each flocculation time, four flocculant dosing strategies were applied, maintaining a total dosage of 15 g/t in all cases, as described in the sedimentation tests (see Section 2.2).

Flocculation kinetic measurements were performed using a Particle Track G400 (Mettler Toledo, Columbus, OH, USA) instrument employing Focused Beam Reflectance Measurement (FBRM) technology. This technique enables in situ, real-time monitoring of changes in the size and concentration of suspended particles. The measurement is based on a probe that scans the suspension using a focused laser beam rotating rapidly at a tangential speed of 2 m/s. When the beam intercepts a particle, it generates a pulse of reflected light whose duration is proportional to the corresponding chord length.

The FBRM system records chord length distributions ranging from 0.25 to 1000 µm across 100 logarithmically spaced channels, with data acquisition occurring every 2 s. Data obtained using the iC FBRM 3.1 software were analyzed in two modes: unweighted distributions and square-weighted distributions. The unweighted mode provides higher resolution for analyzing fine particles and bimodal distributions, while the square-weighted mode is better suited for evaluating the evolution of larger particles.

It should be noted that FBRM provides chord length distributions rather than direct measurements of floc or particle diameter [24]. Therefore, all size-related metrics reported in this study are expressed in chord length and are used for comparative and relative evaluation of floc growth, breakage, and restructuring under different dosing strategies.

### 2.5. Determination of Fractal Dimension and Aggregate Density

The fractal dimension (Df) is a fundamental parameter for analyzing the structural configuration of aggregates formed during sedimentation. This index, which ranges from 1 to 3, provides information on aggregate morphology: values near 1 indicate elongated, open structures, while values near 3 indicate denser, more compact aggregates.

To determine Df, the model proposed by Heath et al. [25] was used, which relates hindered sedimentation velocity to aggregate diameter using Equation (1). In the present study, the aggregate diameter ($d_{agg}$) was approximated with the weighted mean square chord length obtained using FBRM.

(1) $$U_{h}=\frac{d_{agg}^{2}\;g\;\left(\rho_{s}-\rho_{l}\right){\left(\frac{d_{agg}}{d_{p}}\right)}^{Df-3}}{18µ}{\left(1-\varphi_{s}{\left(\frac{d_{agg}}{d_{p}}\right)}^{3-Df}\right)}^{4.65}$$

where $U_{h}$ represents the limited settling rate (m/s), $d_{agg}$ is the aggregate size under the tested conditions (m), and $d_{p}$ is the average diameter of the individual particles (m); $\rho_{s}$ and $\rho_{l}\;$ correspond to the densities of the solid and liquid phases, respectively (kg/m^3^), g is the gravitational acceleration (m/s^2^), μ is the dynamic viscosity of the liquid (N s/m^2^), and $\phi_{s}\;$ is the volumetric solids concentration. The fractal dimension *Df* is obtained by experimentally fitting Equation (1) to the hindered settling rate data. The values of $d_{p}$, $\rho_{s}\;$, $\rho_{l}$, g, μ, and $\phi_{s}$ are considered constant throughout all the tests performed (see Table 2) while $U_{h}$, $d_{agg}$ and Df are variables that depend on the specific conditions of each test.

The fractal dimension was obtained by fitting Equation (1) to the experimentally measured hindered settling velocities using a least squares regression method. The fit was consistent across all tests, with identical fluid and particle properties, allowing for comparison of dosing strategies. Additionally, the apparent density of the aggregates ($\rho_{agg}$) was estimated using the model proposed by Kranenburg [26], which establishes a relationship between $\rho_{agg}$, $\rho_{s}$, $\rho_{l}$; the diameters $d_{agg}$ and $d_{p}$; and the fractal dimension *Df*. This methodology provides a detailed characterization of floc physical properties, enabling more precise analysis of their behavior during sedimentation.

## 3. Results and Discussion

This section organizes and discusses the experimental results to clarify how the timing of flocculant dosage in seawater modulates flocculation performance, both macroscopically and microscopically. First, sedimentation tests are analyzed under different quartz-kaolinite ratios and at different flocculation times. Subsequently, the kinetics of floc formation and breakup are examined using FBRM, enabling a dynamic interpretation of the processes leading up to sedimentation. Next, unweighted and square-weighted size distributions are evaluated to characterize the evolution of the aggregate spectrum under each polymer application scheme. This analysis is complemented by the study of derived structural parameters, such as fractal dimension and bulk density, which provide insight into the flocs’ compactness and internal stability. Finally, zeta potential measurements of quartz and kaolinite in seawater and distilled water, with and without PAM, are presented to contextualize the contribution of the particles’ surface state to the mechanisms of adsorption, bridging, and capture of fines.

### 3.1. Influence of Polymer Dose Sequencing on Settling Kinetics in Seawater

**Figure 3:**
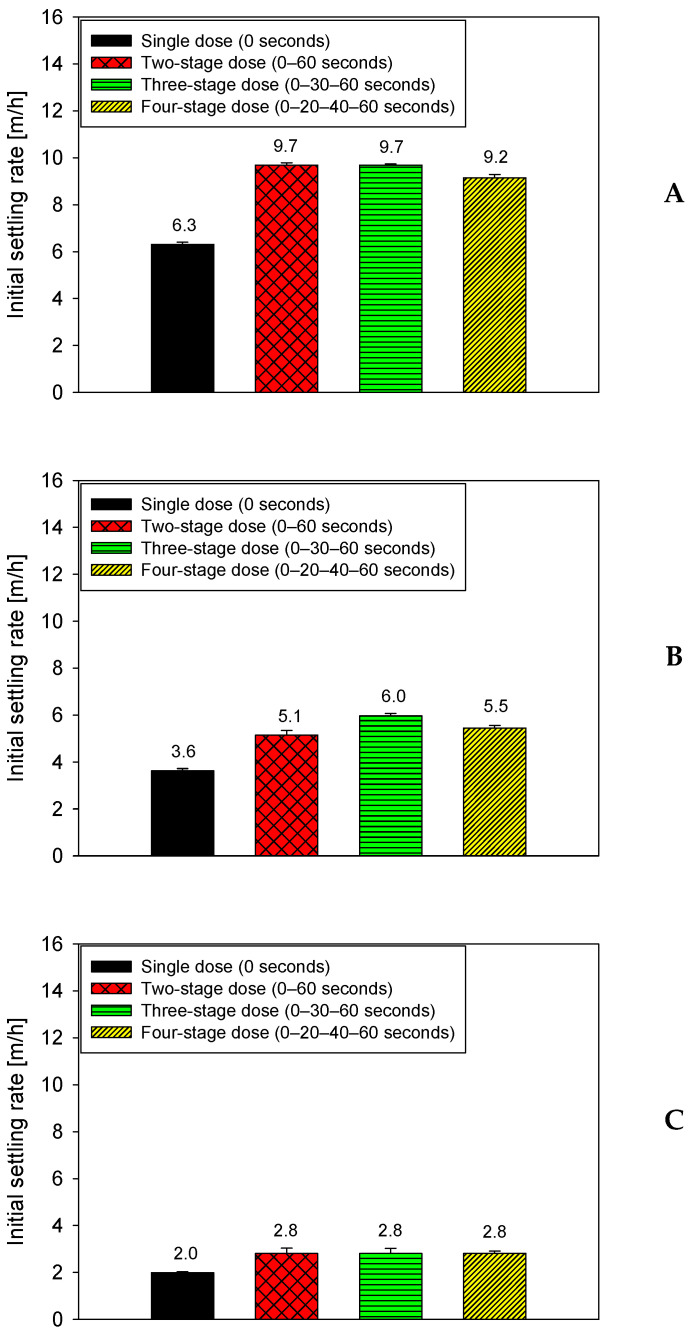
Effect of mineral composition on settling rates in seawater at 15 g/t PAM, 75 s flocculation, and four dosing strategies, pH 7. (**A**): 90–10, (**B**): 80–20, (**C**): 70–30 quartz–kaolinite.

Figure 3 examines the effect of mineral composition on the settling performance of quartz-kaolinite suspensions in seawater under a fixed flocculation time of 75 s. All tests were performed at pH 7 using a total dosage of 15 g/t of anionic PAM (SNF 704), applied using four dosing strategies: a single addition at 0 s and three split schedules (0–60 s, 0–30–60 s, and 0–20–40–60 s). The three graphs correspond to mixtures with the quartz-kaolinite ratios shown in Figure 3A: 90–10, Figure 3B: 80–20, and Figure 3C: 70–30, respectively.

In all three systems evaluated, Figure 3 shows that split application maintains a clear advantage over single-pulse application, even though the increased clay fraction reduces the separation between the strategies. For the 90–10 mix (Figure 3A), multi-pulse application strategies reach speeds of 5.8–6.0 m/h, while single-pulse application strategies drop to 3.3–3.6 m/h. In the 80–20 mix (Figure 3B), split application schemes stabilize at 5.0–5.5 m/h, compared to 3.0–3.2 m/h for single-pulse application. Finally, in the most clayey mix (70–30; Figure 3C), split-application strategies remain between 4.5 and 5.0 m/h, whereas single-pulse application is reduced to 2.5–2.8 m/h. This behavior confirms that, although the relative differences between fractionated schemes decrease with higher kaolin content, due to the greater surface demand and rapid primary coagulation, all stepped strategies mitigate the significant loss of efficiency associated with a single pulse.

**Figure 4:**
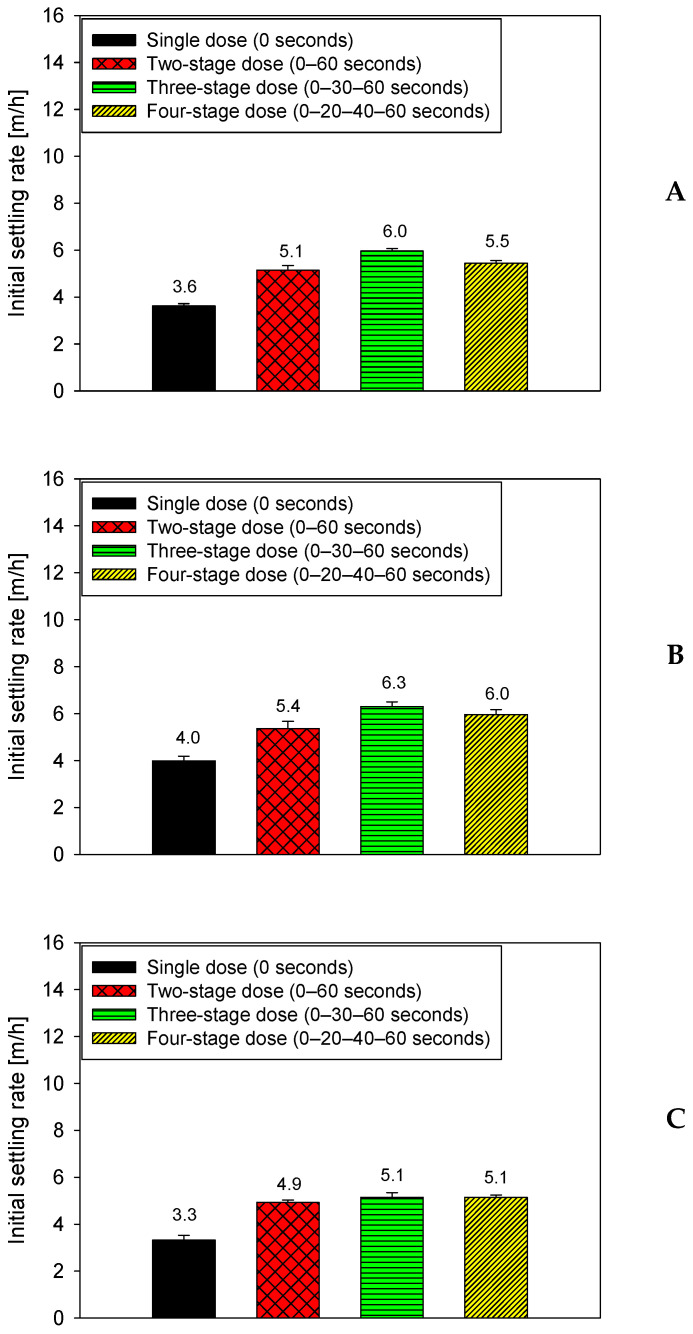
Settling rates in seawater at 15 g/t flocculant dosage for an 80–20 quartz–kaolinite mixture under four dosing strategies, evaluated at three flocculation times: 75 s (**A**), 90 s (**B**), and 105 s (**C**), pH 7.

Figure 4 shows the effect of flocculation time on the settling rate of a suspension containing 80% quartz and 20% kaolin in seawater, maintaining a constant dose of 15 g/t of anionic PAM. The four dosing strategies are applied within the first 60 s, after which three total flocculation times are evaluated: 75 s (A), 90 s (B), and 105 s (C). This figure allows for the specific isolation of the evolution of settling performance after polymer addition is complete.

The results show that the flocculation time after dosing significantly affects system performance. Between 75 and 90 s, all strategies exhibit a significant increase in settling velocity: fractionated dosing schemes increase from approximately 5.0–5.5 m/h to 5.8–6.3 m/h, while single dosing increases from 3.0–3.2 m/h to 3.8–4.0 m/h. This behavior indicates that at 90 s, the flocs have reached an optimal degree of consolidation, with sufficiently developed polymer bridges, but have not yet entered the floc deterioration regime. However, when flocculation is extended to 105 s, a decrease in efficiency is observed: fractional strategies drop to 4.8–5.1 m/h and single-pulse addition falls again to 3.2–3.4 m/h, suggesting internal rearrangements or weakening due to prolonged shear stress. Even so, fractional strategies always maintain a clear advantage over single-pulse addition, particularly at 90 s, when the combination of sequential adsorption and controlled maturation maximizes floc stability and settling capacity.

### 3.2. Real-Time Flocculation Kinetics Under Fractionated Polymer Dosing

**Figure 5:**
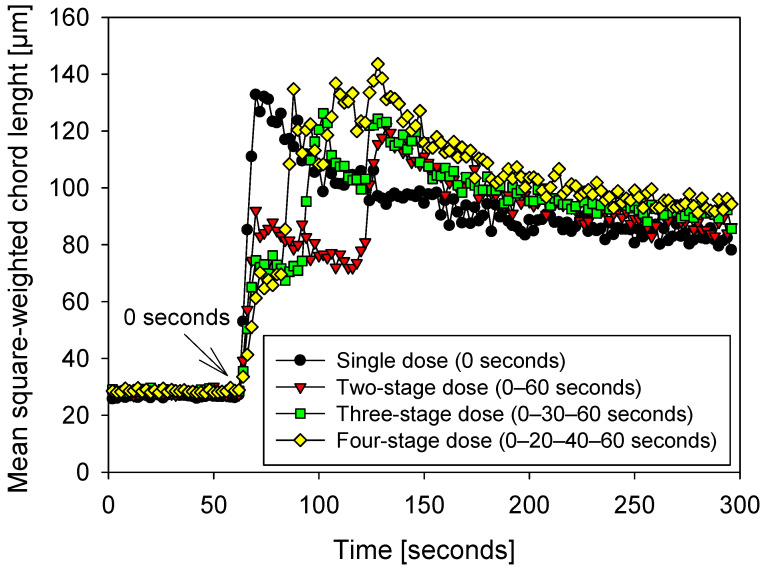
Flocculation kinetics in 15 g/t seawater with four flocculant dosing strategies (0 s, 0–60 s, 0–30–60 s, and 0–20–40–60 s), pH 7.

The FBRM signal in Figure 5 reveals, with two-second resolution, how each dosing strategy modulates the four typical phases of flocculation in seawater:

In the initial aggregation phase (0–≈20 s) after the first injection, corresponding to 60 s on the x-axis, a single dose causes an abrupt increase, with the flocs reaching ~130–140 µm almost immediately. This local overdosing saturates the surfaces and generates large, but probably loosely compacted, flocs. Therefore, their size reduction is more intense than with the other dosing strategies.

During fractional growth (≈20–70 s), the second and third injections (red and green) act as “booster feeding”: the red curve reaches ~120 µm after its 60 s dose, while the green and yellow curves continue to grow to ~130 and >140 µm, respectively. This sequential aggregation facilitates the capture of fines and the resorption of extended chains, resulting in denser, more uniform aggregates.

In the shear rupture phase (≈70–150 s), all curves decrease; the slope is steeper for the single injection, confirming that flocs formed with excess polymer are mechanically weaker. Multistage strategies exhibit increasingly gentler slopes because aggregates form at lower polymer concentrations and, therefore, possess a network of extensible bridges that better resist hydrodynamic deformation.

Finally, at maturation (150–300 s), the values stabilize: ≈80 µm (black), 85–90 µm (red), 90–95 µm (green), and ~100 µm (yellow). The order reflects the structural robustness obtained during growth; moreover, it is consistent with the results obtained for the sedimentation velocities: at 90 s of flocculation, 0–30–60 s is the fastest strategy (6.3 m h^−1^), followed by 0–20–40–60 s (≈6.0 m h^−1^), 0–60 s (5.4 m h^−1^), and single injection (4.0 m h^−1^). Kinetic-sedimentation coherence confirms that peak size alone is insufficient: what matters is the floc’s ability to retain its integrity until the sample is transferred to the sedimentation cylinder.

The peak values, time to peak, and decay slope indicate, respectively, capture efficiency, adsorption kinetics, and resistance to fragmentation. Integrated with the floc density and fractal dimension reported below, they point to an optimal point at three fractions (0–30–60 s): they produce compact aggregates (Df ≈ 2.82) but avoid the over-fragmentation observed when shear is extended to 105 s. The four-fraction strategy matches—or slightly exceeds—the initial size, but its density and sedimentation velocity exhibit marginal—and, in some cases, diminishing—returns.

### 3.3. Size Distribution Analysis of Flocs Formed Under Fractionated Polymer Dosing

**Figure 6:**
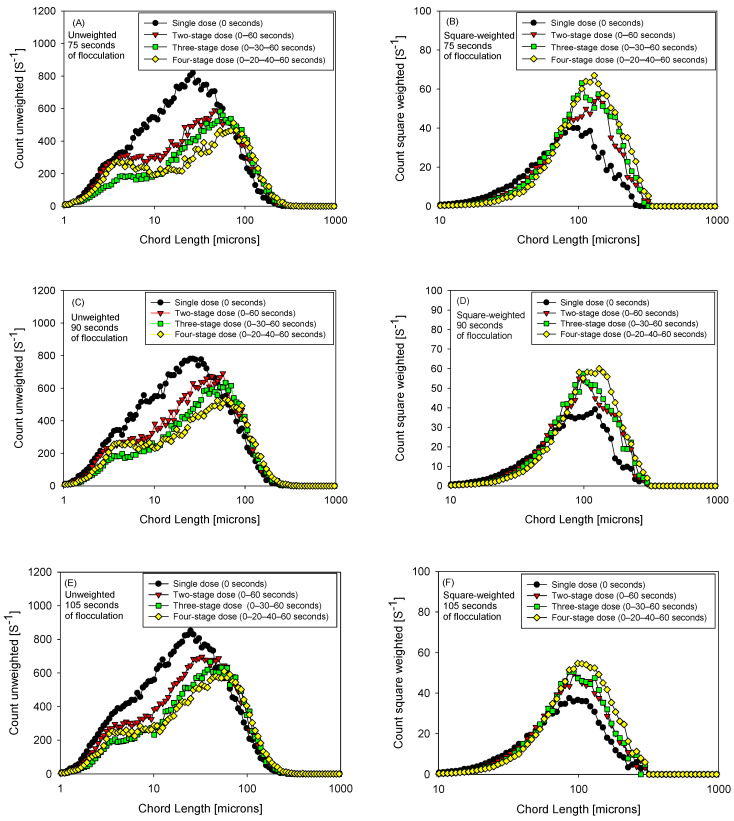
Unweighted (**A**,**C**,**E**) and quadratically weighted (**B**,**D**,**F**) particle size distribution, flocculation 75, 90, and 105 s, four flocculant dosing strategies (0 s, 0–60 s, 0–30–60 s, and 0–20–40–60 s), 15 g/t flocculant, pH 7.

Figure 6 shows, for 75, 90, and 105 s of flocculation, pairs of unweighted (A, C, E) and square-weighted (B, D, F) FBRM distributions superimposing four dosing strategies (1, 2, 3, and 4 pulses; total dose 15 g/t, pH 7). The unweighted curves, which are sensitive to the fine population, show that three pulses (0–30–60 s) more effectively attenuate the first peak < 10 μm—evident in the green curve—indicating lower persistence of fines and a cleaner transition to intermediate classes. Analyzing the unweighted size distribution at 90 s of flocculation (Figure 6C), the three-pulse strategy (green curve) shows a clear attenuation of the first peak, located around 5 µm. In this case, the maximum peak height is slightly above 200 s^−1^. In contrast, the two-pulse (red curve) and four-pulse (yellow curve) strategies exhibit maximum values above 250 s^−1^ in this same region, indicating greater persistence of fine particles. Single-pulse dosing (black curve) shows a significantly higher signal in the sub-10 µm range and a broader distribution, reflecting a dominant fines population and a broader and less well-resolved peak associated with this fraction.

In the square-weighted curves, which emphasize the larger sizes, the lengthening of the right tail and the modal shift are more marked with four pulses (0–20–40–60 s), evidencing the generation of larger flocs (a “heavier” tail) even when some fine residue coexists. Comparing the three times, at 75 s the fractionation already reduces the fine fraction but the growth of large particles is incipient; at 90 s both readings converge on a favorable compromise (minimum at the fines peak and extended tail without overcutting), and at 105 s there are signs of re-fragmentation/reconfiguration: part of the fine fraction reappears in the unweighted and the additional gain in the square-weighted stabilizes or decreases.

Overall, the shape of the distributions suggests a trade-off controlled by the number and spacing of pulses: 3 pulses maximize the capture of fine particles with sufficient structural robustness, while four pulses favor the consolidation of ultra-large particles; this pattern is consistent with previous kinetics and with the performance trends reported for 75–90–105 s in the manuscript.

**Figure 7:**
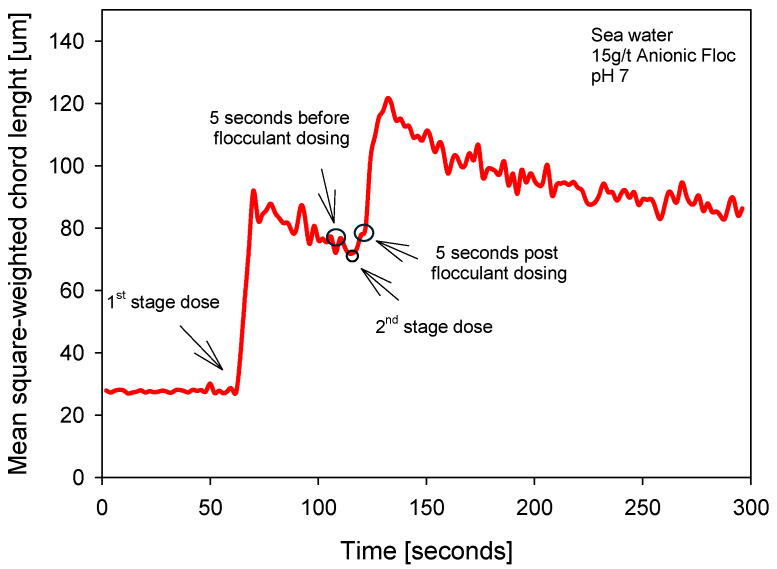
Flocculation kinetics two-stage flocculant aggregation strategy (0–60 s).

Figure 7 shows the flocculation kinetics in seawater (pH 7; 15 g/t) with two dosing steps, exhibiting a clear growth–relaxation–growth–relaxation sequence. After a stable baseline mean size (measured as the square of the weighted chord length), the first dose triggers an abrupt jump with a short time to maximum and a subsequent relaxation to an intermediate value. The ascending segment reflects growth driven by adsorption and bridging onto available particles; the descending slope reflects breakage and reconfiguration under shear. In the five-second window before the second dose, a partially mature state of the suspension is observed, which serves as a reference for quantifying the impact of the second pulse. The second dose generates a new overshoot, more pronounced than the previous natural variation, followed by relaxation toward a quasi-steady state.

**Figure 8:**
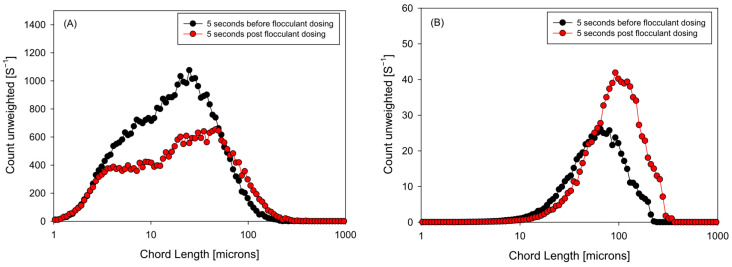
Unweighted (**A**) and square weighted (**B**) chord length distribution for two-stage flocculant aggregation strategy (0–60 s).

Figure 8 contrasts the size distributions in these two windows, using the unweighted readout (more sensitive to fines) and the square-weighted readout (which emphasizes large flocs). In the unweighted readout, the state before the second dose retains a first mode associated with a fine population; five seconds after dosing, the curve shows a relative reduction in this first peak and a shift toward intermediate classes, indicating that fines are captured in the brief interval between pulses. In the square-weighted readout, the pre- and post-dose contrast reveals an immediate increase in floc size—greater contribution from high chord lengths—consistent with the overshoot observed in the kinetics: the additional polymer acts on existing agglomerates, favoring bridging and generating flocs of larger equivalent size.

### 3.4. Fractal Dimension and Aggregate Density Under Fractionated Polymer Dosing

**Figure 9:**
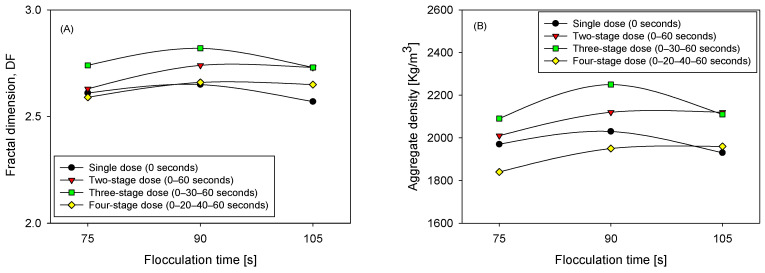
Fractal dimension (**A**) and aggregate density (**B**), for three flocculation times (75, 90, and 105 s) and four flocculant dosing strategies (0 s, 0–60 s, 0–30–60 s, and 0–20–40–60 s), pH 7.

Although polymer chain contraction occurs in high ionic strength environments, the fractal model remains applicable for comparative analysis, as the same saline conditions and polymer chemical composition were maintained across all experiments. In this context, the fractal dimension is interpreted as a relative descriptor of aggregate compactness, rather than an absolute structural parameter, consistent with previous applications of fractal models in saline systems [27]. Figure 9 illustrates that the final structure of the agglomerates is clearly dependent on both the dosing sequence and the total flocculation time. In comparative terms, the most consistent pattern is that three pulses tend to exhibit the highest values of fractal dimension and apparent density in the intermediate time, indicating more compact agglomerates with lower effective porosity; one pulse is systematically associated with less dense structures and less consolidation; two pulses offer partial improvements compared to one pulse, but without reaching the level of compaction observed with three; four pulses can approach the maximum in some cases, although with greater dispersion and without the sustained reduction in fines that characterizes the three-stage strategy.

This hierarchy is accentuated at 90 s, when the balance between growth and shear strength favors more cohesive networks. At 75 s, maturation remains incomplete, and the structural indicators are more modest. At 105 s, signs of reconfiguration or fragmentation appear, reducing the structural gains. The consistency with two-stage kinetics is straightforward: a second, and especially a third, injection into a partially structured matrix enhances the effective bridging and consolidation of particle networks, increasing both the fractal dimension and density. However, a fourth injection and prolonged exposure to shear can induce rearrangements that limit the net benefit.

The reading is also compatible with the distributions: when the fine fraction decreases steadily, and a sufficiently extended tail is maintained, the flocs become more compact and denser.

It is important to note that the increased compaction and aggregate density observed under fractional dosing in this study contrast with results reported for biological systems, such as those of Zhang et al. [13], where intermittent dosing led to larger, more structurally open flocs. The intrinsic characteristics of the systems evaluated explain this difference. In mineral suspensions, such as the quartz-kaolinite system analyzed, flocculation is dominated by polymeric bridging mechanisms between rigid particles, allowing the sequential addition of anionic PAM to promote internal reconfiguration, pore closure, and increased fractal dimension under controlled shear conditions. In contrast, biological systems involve deformable cells, matrices of extracellular polymeric substances (EPS), and predominantly cationic flocculants, in which charge neutralization and loose network formation limit structural compaction. Additionally, the milder mixing conditions, typical of microalgae flocculation, reduce the possibility of densifying restructuring.

### 3.5. Zeta Potential Analysis Under Seawater Conditions and Polymer Addition

**Figure 10:**
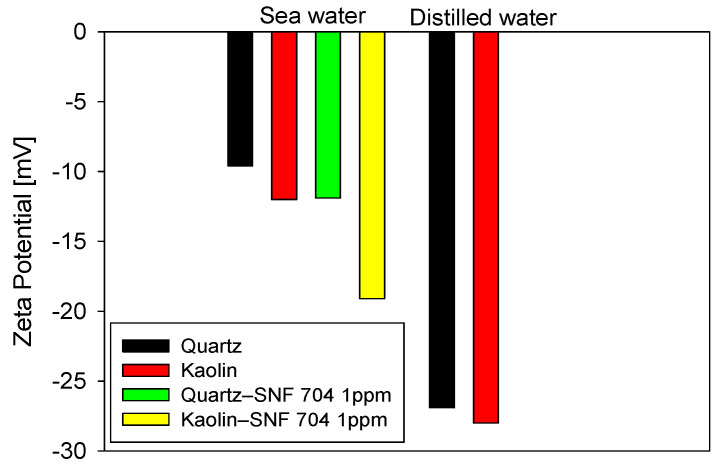
Zeta potential of quartz and kaolinite in seawater and distilled water, with and without 1 ppm of PAM (SNF 705).

The results in Figure 10 show that adding 1 ppm anionic PAM (SNF 705) appreciably modifies the zeta potential of both minerals, with magnitudes that vary with mineral surface. In distilled water, both quartz and kaolinite exhibit highly negative values (≈−25 mV), reflecting an extended double layer and a regime dominated by electrostatic repulsion. In seawater, the strong compression of the double layer shifts the values toward more positive levels (≈−5 to −15 mV). However, even under these conditions of high ionic strength, the addition of the flocculant produces a further decrease in the magnitude of the potential, less pronounced in quartz and clearly more so in kaolin. The differential response observed between kaolinite and quartz to the addition of acrylamide polymers (AMPs) can be explained by the structural and chemical characteristics of their surfaces under saline conditions. Unlike quartz, whose siloxane surface has a low density of reactive sites, kaolinite exhibits basal faces and edges rich in aluminol and silanol groups, with pH-dependent charge. Molecular-scale studies have shown that, in saline solutions, the adsorption of acrylamide polymers onto kaolinite is dominated by the formation of cationic bridges mediated by monovalent and divalent cations (Na^+^, Ca^2+^, and Mg^2+^) between the polymer’s carboxylate groups and the mineral’s reactive sites, surpassing hydrogen bonding interactions in frequency and stability, especially at crystal edges [28]. These simulations also show that these mechanisms are significantly less effective on quartz surfaces, which explains the greater polymer-kaolinite affinity at high ionic strength. This behavior is reflected macroscopically in the zeta potential results, where kaolinite shows a more pronounced shift after the addition of PAM and correlates directly with the greater fines capture observed in the unweighted FBRM distributions.

The shift in zeta potential confirms that, despite the salinity, the polymer not only acts by physical bridging but also exerts a measurable effect on surface charge, especially in the clay fraction. This observation is consistent with previously reported kinetic and structural results, which show that kaolin accounts for a large part of the fine fraction. Therefore, its greater affinity for the polymer contributes decisively to the capture efficiency of fractional-dosing strategies.

## 4. Conclusions

This study demonstrates that fractional dosing of anionic polyacrylamide in seawater is an effective strategy for controlling floc growth, structural stability, and settling performance in quartz-kaolinite suspensions. Compared to single-pulse dosing, all fractional strategies consistently improved settling rates, reduced fines persistence, and increased aggregate robustness, confirming that temporal control of polymer availability is a key variable in the flocculation of saline mineral systems.

The results show that a flocculation time of 90 s represents an optimal operating window, maximizing floc growth and structural consolidation without entering a regime dominated by reconfiguration or shear-induced deterioration. Shorter times lead to incomplete maturation and less compact structures, while prolonged exposure to shear reduces the structural benefits gained during the growth stage. Within this optimal range, the three-pulse strategy (0–30–60 s) provides the best balance between polymer availability, effective bridging, and resistance to hydrodynamic stress. Although greater temporal fragmentation in four pulses can increase the initial aggregate size, it does not yield additional improvements in fractal dimension or bulk density. It introduces diminishing returns as greater exposure of partially formed flocs to shear.

The integration of FBRM kinetic results, size distribution analysis, fractal dimension, aggregate density, and zeta potential indicates that the effectiveness of fractionated dosing is governed by the interplay among polymer availability, the surface reactivity of clay minerals, and the system’s shear history. In particular, the greater affinity of anionic PAM for kaolinite under high salinity conditions—mediated by cationic bridging mechanisms—confirms that the clay fraction controls fines capture and the overall efficiency of the flocculation process. These results demonstrate that fractional dosing should not be considered merely a reagent management practice but a structural control tool capable of modulating floc architecture and stability. From an industrial perspective, this mechanistic understanding provides a solid foundation for optimizing flocculant injection strategies in tailings thickening operations using seawater or other high-ionic-strength waters.

## Figures and Tables

**Table 1:** Composition of synthetic seawater.

Component	Concentration (g/L)
NaCl	24.53
MgCl_2_·6H_2_O	11.10
Na_2_SO_4_	4.09
CaCl_2_	1.16
KCl	0.69
NaHCO_3_	0.20
KBr	0.10
H_3_BO_3_	0.03

**Table 2:** Parameters used for calculating fractal dimensions and aggregate density.

Parameter	Values
$d_{p}$ (µm)	27.2
$\rho_{solid}$ (kgm^−3^)	2600
$\rho_{SW}$ (kgm^−3^)	1025
g (ms^−2^)	9.8
${µ}_{SW}$ (Nsm^−2^)	0.001077
$\varphi_{s}$	0.042

## Data Availability

The original contributions presented in this study are included in the article. Further inquiries can be directed to the corresponding author.
